# Congenital Disorders of Glycosylation: A Multi-genetic Disease Family with Multiple Subcellular Locations

**DOI:** 10.34763/jmotherandchild.20202402si.2005.000004

**Published:** 2020-11-10

**Authors:** Jaak Jaeken

**Affiliations:** 1 Department of Development and Regeneration, Center for Metabolic Diseases, University Hospital Gasthuisberg, KU Leuven, Leuven, Belgium

**Keywords:** congenital disorders of glycosylation, protein glycosylation defects, N-glycosylation disorders, O-glycosylation disorders, cytosolic CDG, endoplasmic reticulum-related CDG, ER-related CDG, ER–Golgi intermediate compartment (ERGIC) CDG, plasma membrane, sarcolemmal membrane

## Abstract

This review discusses a selection of congenital disorders of glycosylation that show peculiar features, such as an unusual presentation, different phenotypes, a novel biochemical/genetic mechanism, a relatively high frequency or a relatively efficient treatment.

## Introduction

Congenital disorders of glycosylation (CDG) are genetic defects in the synthesis of glycans and their attachment to proteins and lipids to form glycoproteins and glycolipids, respectively. Protein glycosylation defects comprise protein *N*-glycosylation and *O*-glycosylation disorders. I am not aware of defects in *C*-glycosylation. The number of reported CDG has rapidly increased and amounts to at least 130. This group of metabolic diseases is very special in that, contrary to most other metabolic diseases that are limited to one organelle, these have a multi-subcellular distribution. Defects have been found in the cytosol, the endoplasmic reticulum (ER), the ER–Golgi intermediate compartment (ERGIC), the Golgi apparatus, the sarcolemmal membrane and the plasma membrane.

The large majority of CDG are inherited in an autosomal recessive manner. About 15 CDG show an autosomal dominant or X-linked inheritance, and, interestingly, a few show one or the other inheritance depending on their variant(s) (see, e.g. POFUT1-CDG and COG4-CDG below). This review aims to discuss, mainly from a clinical point of view, a selection of CDG with peculiar features, e.g. an unusual presentation, different phenotypes, a novel biochemical/genetic mechanism, a relatively high frequency or a rather efficient treatment. See recent reviews for complementary information (1, 2, 3, 4).

## Cytosolic CDG

### PMM2-CDG

Phosphomannomutase 2 (PMM2) deficiency is the first clinically described CDG. It is the most frequent protein *N*-glycosylation disorder, with at least 900 reported patients. The other known *N*-glycosylation disorders have been reported in <100 patients. As in most other CDG, there is a predominant neurological involvement, but almost any other organ/system can be affected. Peculiar features are inverted nipples and unusual subcutaneous fat pads. The combination and severity of signs and symptoms can vary widely, even between affected siblings. In the large majority of patients, serum transferrin (Tf) isoelectrofocusing (IEF) shows a type 1 pattern (decreased tetrasialo-Tf and increased disialo-Tf and asialo-Tf). An excellent overview is provided by Grünewald (5). A remarkable variant is the *PMM2* promoter defect c.-167G>T, homozygous or in *trans* with *PMM2*-coding variants, resulting in a very restricted phenotype of polycystic kidney disease (and liver cysts in half of them) and hyperinsulinaemic hypoglycaemia (6). Treatment of PMM2-CDG is symptomatic, and trials with liposomal mannose 1-phosphate and chaperones are underway; for a review, see Verheijen et al. (7). Recently, acetazolamide has been shown to be effective in the treatment of the (fairly disturbing) motor cerebellar syndrome of this CDG (8).

### MPI-CDG

Mannosephosphate isomerase (MPI) deficiency belongs to the most common *N*-glycosylation disorders, together with PMM2-CDG, ALG1-CDG, ALG6-CDG, SRD5A3-CDG and SLC35A2-CDG. It is a defect in the very first step of the mannose pathway and one of the few *N*-glycosylation disorders without neurological involvement. It is a usually lethal hepato-intestinal disorder, but oral mannose administration can restore the pathway. It is one of the very few CDG with a rather efficient basic treatment. Some patients suffer from severe side effects on mannose intake, and they can be saved by liver transplantation; see Jaeken et al. (9) for a review.

### GMPPA-CDG

Guanosine diphosphate-mannose pyrophosphorylase A (GMPPA) deficiency is known as alacrima, achalasia and mental retardation (AAMR) syndrome. It resembles the triple A syndrome, but patients with AAMR do not have adrenal insufficiency. Remarkably, there is no evidence of abnormal *N*-glycosylation in these patients (10).

### GMPPB-CDG

Guanosine-diphosphate-mannose pyrophosphorylase B (GMPPB) deficiency is clinically very different from, and more frequent than, GMPPA-CDG. It is associated with a wide neurological spectrum, ranging from Walker-Warburg syndrome to pseudo-metabolic myopathy, and even congenital myasthenic syndromes. Less frequent features are the asymptomatic increase of serum creatine kinase, arthrogryposis, congenital clubfoot, seizures and autism spectrum disorder. As in GMPPA-CDG patients, *N*-glycosylation seems to be preserved (11).

### CAD-CDG

CAD encodes a multi-functional enzyme involved in de novo pyrimidine, and hence uridine diphosphate (UDP) and cytosine monophosphate (CMP), biosynthesis. Its deficiency causes a severe syndrome with global intellectual disability, early infantile epilepsy and dyserythropoietic anaemia. Nearly all UDP-activated sugars, which are donors for glycosylation, have been reported to be decreased, but the serum Tf IEF is normal. Promising results have been obtained by oral uridine supplementation in two children, leading to immediate cessation of seizures and significant improvement of psychomotor development (12).

### PGM1-CDG

Phosphoglucomutase 1 (PGM1) deficiency is a multi-system CDG that leads to decreased *N*-glycan synthesis in the ER and decreased galactosylation in the Golgi. It is therefore a ‘dual CDG’ (CDG-I/II) visualised as a type 2 pattern on serum Tf IEF. Galactosaemia is the only other ‘dual’ glycosylation disorder known. Patients show a unique combination of split uvula/palate and muscle, liver, cardiac, endocrine and coagulation factor involvement. The hypogalactosylation suggests treatment with oral galactose. This treatment improves hypoglycaemia, hypertransaminasaemia, and glycosylation parameters (partially). Evidence has been provided that galactose is effective among others through the replenishment of galactose-1-phosphate, UDP-glucose and UDP-galactose (13).

### PGM3-CDG

Phosphoglucomutase 3 (PGM3) deficiency is a disorder of *N*-acetylglucosamine biosynthesis. It shows a broad phenotype with predominant immunological involvement: recurrent infections, atopic disease, psychomotor disability, failure to thrive, skeletal abnormalities, eosinophilia, high levels of serum immunoglobulin E (IgE) (hyper-IgE) and decreased counts of cluster of differentiation 4 (CD4) T-cells, besides many other signs/symptoms present in a minority of the patients. Unexpectedly, serum Tf and apolipoprotein C (apoC-III) IEF are normal. Bone marrow transplantation and haematopoietic stem cell transplantation are therapeutic options; for a review, see Jaeken et al. (14).

### EOGT-CDG

Adams-Oliver syndrome is characterised by aplasia cutis congenital of the scalp and is mostly associated with terminal transverse limb defects, besides other abnormalities (neurological, cardiovascular and ocular). There are at least six genes for this syndrome and two modes of inheritance. Autosomal recessive Adams-Oliver syndrome is caused by variants in the *DOCK6* and *EOGT* genes. The latter encodes an *N*-acetyl glucosamine transferase, which links *N*-acetyl glucosamine to threonine hydroxyl moieties within the epidermal growth factor (EGF)-like domain of Notch receptors. This protein is present in both the nucleus and the cytosol. Its defect has been labelled as Adams-Oliver syndrome 4 (15).

### OGT-CDG

While EOGT-CDG is caused by EGF-domain-specific *O*-GlcNAc transferase (EOGT) defects, OGT-CDG is associated with defects in an X-linked OGT (in the nucleus and the cytosol) that catalyses the covalent attachment of *N*-acetylglucosamine to serine or threonine hydroxyl moieties of a very large number of nucleocytoplasmic proteins. In addition, the phenotype, mainly intellectual disability, is completely different from the aforementioned CDG (16).

### DHDDS-CDG

Dehydrodolichol diphosphate synthase (DHDDS) deficiency is one of the three known defects in the synthesis of dolichol phosphate, the carrier of the glycan intermediates in the ER. It causes either autosomal recessive non-syndromic retinitis pigmentosa (17) or a de novo encephalopathy comprising epilepsy and other movement disorders, hypotonia and developmental and intellectual disability (18).

## ER-related CDG

### ALG1-CDG

Mannosyltransferase 1 deficiency is characterised by a predominant neurological involvement: mostly severe intellectual developmental disability and hypotonia. A majority of patients show dysmorphism, microcephaly, intractable epilepsy, visual disturbances, tremor, ataxia, severe infections and brain abnormalities. Life expectancy ranges from 1 day to >20 years. Serum tTf IEF shows a type 1 pattern, and recently, a novel biomarker was detected in these patients, namely the protein-linked xeno-tetrasaccharide NeuAc–Gal– GlcNAc_2_ (19).

### ALG6-CDG

Glucosyltransferase 1 deficiency is mostly characterised by a mild to moderately severe neurological disorder and feeding problems. Many other symptoms have been reported, the most uncommon being limb anomalies such as brachytelephalangy and short arms. For an unknown reason, blood glycoproteins such as clotting factors XI, antithrombin, protein C and protein S are unusually low, as is serum cholesterol; for a review, see Morava et al. (20).

### ALG9-CDG

Mannosyltransferase 7/9 is one of the three ‘dual’ mannosyltransferases (together with ALG2 and ALG11) that attaches two mannose residues to the growing ER glycan. Its deficiency causes one of two phenotypes: a syndrome with failure to thrive, dysmorphism, epilepsy and hepatic and/or renal cysts; and on the other hand, a lethal skeletal dysplasia in affected foetuses (21). Moreover, *ALG9* mutation carriers can develop kidney and liver cysts (22).

### DPAGT1-CDG

UDP–GlcNAc:Dol–P–GlcNAc–P transferase deficiency presents as one of two different phenotypes: a usually severe encephalopathy with early death in the context of a multi-system disorder, or a congenital myasthenic syndrome. A few cases of adults with a milder, stable disease, have been reported. Serum Tf IEF shows a type 1 pattern in all patients with multi-system presentation but in only half of the patients with congenital myasthenic syndrome. Patients with the latter presentation responded favourably to acetylcholinesterase inhibitors such as pyridostigmine; see review by Ng et al. (23).

### PIGM-CDG

Phosphatidylinositol glycan anchor biosynthesis class M protein transfers the first mannose from dolichol phosphate mannose to phosphatidylinositol, initiating the synthesis of the trimannosyl core of the glycosylphosphatidylinositol (GPI) membrane anchor. Deficient patients show a unique syndrome of portal/cerebrovascular vein thrombosis and absence seizures. They have the same *PIGM* promoter variant. This is the third reported glycosylation gene with a promoter variant besides *PMM2* and *XYLT1*. Decreased histone acetylation has been found at this promoter. Administration of sodium butyrate, which is a histone deacetylase inhibitor, results in significant clinical improvement; reviewed by Pode-Shakked et al. (24).

### DOLK-CDG

Dolichol kinase catalyses the final step in the synthesis of dolichol phosphate. Its deficiency causes an unusual association of symptoms, mainly dilated cardiomyopathy and ichthyosis. The cardiopathy is apparently isolated in some patients. Half of the reported patients died (at 8 days to 11 years). In some patients, there is neurological, hepatic and/or endocrinological involvement. An unusual symptom is truncation of distal phalanges. Cardiac transplantation has been performed in three patients, and two of them survived; see review by Rush et al. (25).

### POFUT1-CDG

Protein *O*-fucosyltransferase 1 (POFUT1) adds *O*-fucose to EGF-like repeats on about 100 mammalian proteins, including Notch receptors. Haplo-insufficiency of the corresponding gene causes generalised Dowling-Degos disease 2, a reticulate pigment skin disorder (26). However, POFUT1 deficiency is also linked to a completely different syndrome comprising developmental disability, microcephaly, cardiopathy and liver disease, due to a homozygous variant (27).

## ERGIC CDG

### SEC23B-CDG

SEC23B is a COPII component. Its deficiency affects only the red blood cell lineage, causing ineffective erythropoiesis, haemolysis, erythroblast morphological abnormalities, hypoglycosylation of some red blood cell proteins (particularly band 3) and secondary abnormalities such as splenomegaly (28). It is one of the few CDG affecting only one organ/tissue. Other examples are EXT1-CDG (cartilage involvement), LFNG-CDG (vertebral column), POFUT1-CDG (skin), PRKCSH-CDG (liver), ST3GAL3-CDG and TUSC3-CDG (brain).

## Golgi CDG

### EXT1/2-CDG

The protein complex exostosin-1/exostosin-2 catalyses the attachment of glucuronic acid and *N*-acetylglucosamine during the synthesis of heparan sulphate. Deficiency of one of these proteins results in the dominantly inherited multiple exostoses. These are cartilage-capped tumours or osteochondromas on the ends of long bones, often present at birth. Complications may arise from the compression of peripheral nerves or blood vessels, and in a small percentage, there is malignant degeneration. In 2015, a novel and remarkable phenotype of EXT2-CDG was reported without exostoses and with an autosomal recessive inheritance. This old-order Mennonite family showed a seizures–scoliosis–macrocephaly syndrome, besides other features (29).

### GALNT3-CDG

This defect of the polypeptide *N*-acetylgalactosaminyltransferase-3 causes one of two syndromes: hyperphosphataemic familial tumoral calcinosis (HFTC) or the hyperphosphataemic hyperostosis syndrome (HHS). It is a disturbance in the hormonal regulation of serum phosphate levels due to deficient *O*-glycosylation of fibroblast growth factor 23, inactivating this phosphaturic protein. The phenotype of HFTC consists of recurrent, painful calcified subcutaneous masses, and that of HHS consists of episodes of diaphysitis and cortical hyperostosis. Both phenotypes can occur in the same family; reviewed by Jaeken et al. (30).

### SLC35A2-CDG

*SLC35A2* encodes an X-linked protein that transports UDP-galactose from the cytosol to the lumen of the Golgi and the ER. Among the 70 reported patients with SLC35A2-CDG, only nine are males. Epileptic encephalopathy, including psychomotor disability, is the predominant phenotype. In half or less of the patients, microcephaly, ocular/visual abnormalities, a Rett syndrome-like phenotype, cerebral and cerebellar abnormalities, skeletal abnormalities and other symptoms have been reported. Most of these patients have been reported to show a normal serum Tf IEF. In the others, a persisting or transient type 2 pattern is seen. In the large majority of patients, a de novo variant has been observed (31). Galactose supplementation restores glycosylation and improves seizure control (32). Interestingly, brain somatic variants have been found to underlie intractable epilepsy with focal cortical malformations. This is the first reported somatic CDG (33).

### COG4-CDG

The conserved oligomeric Golgi (COG) complex, involved in vesicular transport, is composed of eight subunits distributed between two lobes: lobe A (COG1–4) and lobe B (COG5–8). Defects have been reported in each lobe except COG3. COG4-CDG is caused by biallelic loss-of-function variants and is mainly characterised by epileptic encephalopathy. In 2018, Ferreira et al. (34) showed that a novel heterozygous de novo COG4 variant is responsible for the Saul-Wilson syndrome described in 1990, a rare skeletal dysplasia with dwarfism, facial dysmorphism (progeria-like), hearing loss, cataracts and developmental delay. Contrary to the type 2 serum Tf IEF pattern in ‘classical’ COG4-CDG, this variant shows a normal Tf IEF pattern.

### XYLT1-CDG

XYLT1 deficiency causes Desbuquois dysplasia type 2, a skeletal disorder comprising short stature, dislocation of the large joints, flat face and prominent eyes (35). In 2019, LaCroix et al. identified a GGC repeat expansion and exon 1 methylation of *XYLT1* as a common pathogenic variant in Baratela-Scott syndrome. The latter resembles Desbuquois dysplasia type 2 but also shows developmental disability (36). This is the first CDG associated with epigenetic variants. Epigenetic variants are expected to be important but largely unexplored causes of CDG.

### MAN1B1-CDG

The *MAN1B1* gene encodes Golgi mannosyl-oligosaccharide α-1,2-mannosidase, which is involved in the maturation of N-linked glycans in the secretory pathway. It catalyses the removal of the terminal mannose residue from the middle branch of Man9GlcNAc2, thereby generating Man8GlcNAc2 isomer B. Its impairment results mostly in a relatively mild syndrome comprising developmental/intellectual disability, hypotonia, facial dysmorphism and, in the majority of the patients, truncal obesity too. It is the only known CDG in which truncal obesity is part of the phenotype (37).

## Plasma membrane CDG

### SLC39A8-CDG

This CDG disrupts the function of ZIP8, a major manganese transport protein, thus causing manganese deficiency. The phenotype mainly comprises cranial synostoses with lacunar skull, severe psychomotor disability, epilepsy, vision and hearing impairment, and cerebral and cerebellar atrophy. Malfunction of the manganese-dependent β-1,4-galactosyltransferase causes hypogalactosylation, and galactose supplementation has been reported to have a favourable effect on both clinical features and glycosylation in a severely affected child. Two children treated with oral MnSO_4_ have reportedly shown considerable clinical improvement and normalisation of enzymatic activities (38).

## Sarcolemmal membrane CDG

### TMEM5-CDG

TMEM5 is a ribitol β1,4-xylosyltransferase that transfers xylose to the ribitol 5-phosphate tandem in the *O*-mannosylglycan of α-dystroglycan. TMEM5 deficiency is one of the approximately 18 known dystroglycanopathies. These are autosomal recessive diseases characterised by cerebral, ocular and muscular defects (congenital muscular dystrophies). TMEM5 variants are also frequently associated with neural tube defects and gonadal dysgenesis. Recently, a 15-year-old boy has been reported with congenital muscular dystrophy, increased serum creatine kinase and nearly-absent muscular α-dystroglycan. Contrary to reported patients, no brain or eye defects have been reported in this boy (39). This broadens the clinical spectrum of this CDG, and more generally, illustrates the very broad phenotypic spectrum that is present in many CDG.

## Conclusion

No other metabolic disease family presents such a broad spectrum of signs and symptoms, as well as disease severity, as the CDG. A few CDG show very different phenotypes depending on the variant involved, and on the other hand, there are several CDG phenotypes caused by different genetic defects. Regarding treatment, MPI-CDG is still the only rather efficiently treatable CDG (oral mannose). A few other CDG partially respond to nutritional therapy (fucose, galactose, manganese, ketogenic diet and uridine), medication (acetazolamide) or transplantation (heart, liver and haematopoietic stem cells). For PMM2-CDG, treatments with chaperones, morpholino oligonucleotides, mannose-1-phosphate encapsulated in liposomes and gene therapy are under development. The number of CDG continues to rise exponentially mainly due to next-generation sequencing.
